# Combination of long- and short-axis alar sacroplasty techniques under fluoroscopic guidance for osteoporotic sacral insufficiency fracture

**DOI:** 10.1186/s13018-021-02409-2

**Published:** 2021-04-17

**Authors:** Feng-Chen Kao, Yao-Chun Hsu, Tzu-Shan Chen, Pao-Hsin Liu, Yuan-Kun Tu

**Affiliations:** 1 Department of Orthopedics, E-Da Hospital, Kaohsiung, Taiwan; 2 Department of Orthopedics, Dachang Hospital, Kaohsiung, Taiwan; 3 School of Medicine,College of Medicine, I-Shou University, Kaohsiung, Taiwan; 4 Division of Gastroenterology and Hepatology, E-Da Hospital, Kaohsiung, Taiwan; 5 Department of Medical Research, E-Da Hospital, Kaohsiung, Taiwan; 6 Department of Medical Imaging and Radiological Sciences, College of Medicine, I-Shou University, Kaohsiung, Taiwan; 7 Department of Biomedical Engineering, I-Shou University, Kaohsiung, Taiwan

**Keywords:** Sacral insufficiency fracture, SIF, Sacroplasty, Osteoporosis

## Abstract

**Background:**

Sacral insufficiency fracture (SIF) is rarer than osteoporotic vertebral compression fracture that occurs at other levels of the thoracolumbar spine. Percutaneous sacroplasty can effectively relieve pain and improve mobility. Several sacroplasty-based techniques have been reported to date.

Sacroplasty is often performed with computed tomography-guided cannula placement, which is time intensive and results in greater radiation exposure than that resulting from fluoroscopy. Herein, we report our preliminary experience with a combination of long- and short-axis alar sacroplasty techniques under fluoroscopic guidance for osteoporotic SIFs.

**Methods:**

We retrospectively reviewed 44 consecutive patients with symptomatic osteoporotic SIFs who underwent alar sacroplasty between January 2013 and February 2020. The study group comprised 19 patients who underwent a combination of long- and short-axis alar sacroplasty techniques under fluoroscopic guidance. The control group comprised the remaining 25 patients who underwent short-axis alar sacroplasty under fluoroscopic guidance. Visual analog scale (VAS) scores, operation times, injected cement volumes, and postoperative complications were recorded.

**Results:**

The VAS score for pain decreased in both groups; however, no significant difference was noted between the study and control groups in injected cement volume (3.55 ± 0.96 vs 2.94 ± 0.89 mL). The operation time was longer in the study group than in the control group (32 ± 7.1 vs 28.04 ± 4.99 min; *P* = 0.046). No major complications were noted.

**Conclusion:**

A combination of long- and short-axis alar sacroplasty techniques can be effectively performed under fluoroscopic guidance for osteoporotic SIFs.

## Introduction

**Fig. 1:**
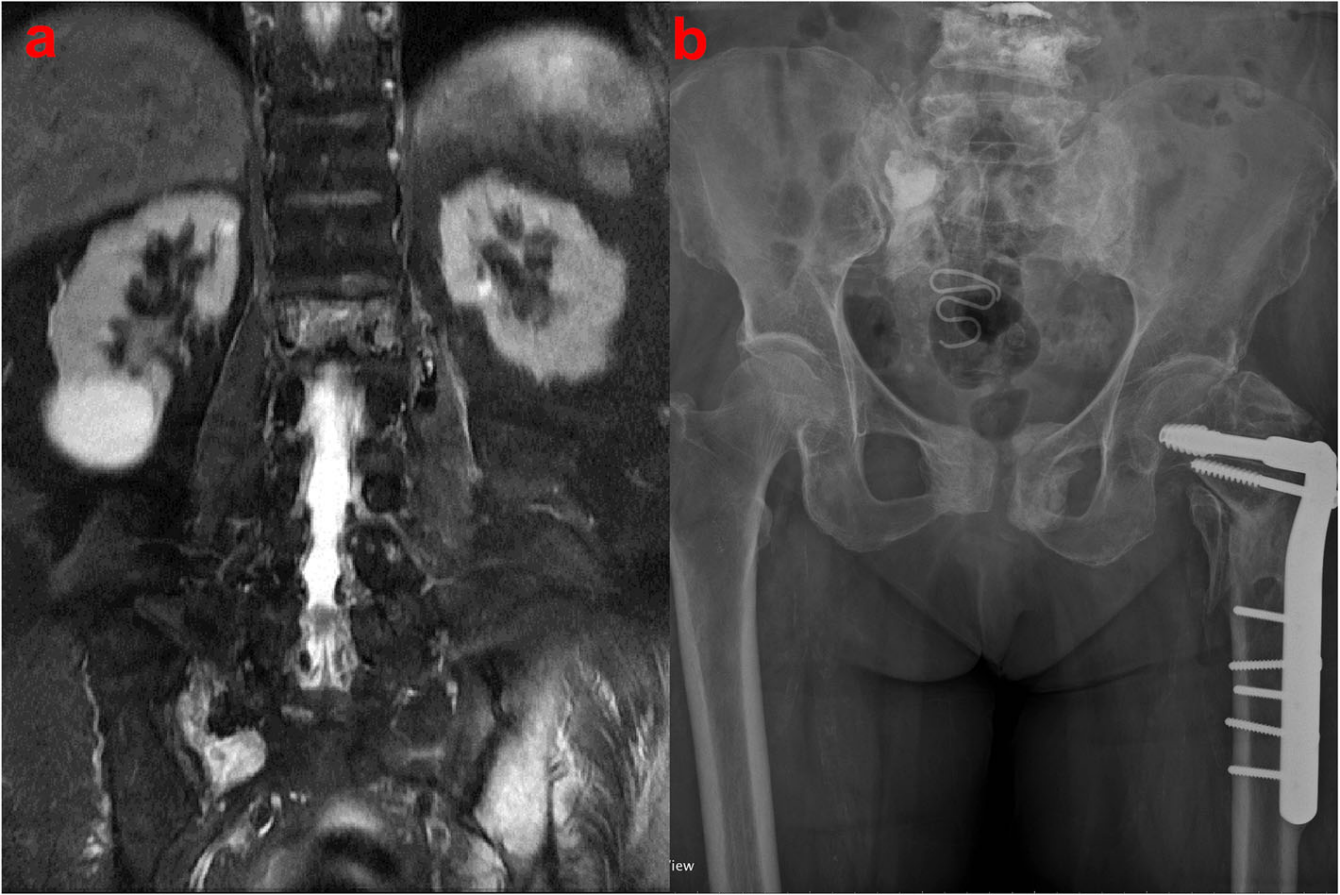
**a** Coronal section of spinal magnetic resonance imaging (MRI) indicating sacral insufficiency fracture involving the right alar area in an 87-year-old female patient. **b** Pelvis anteroposterior view depicting filling in the right alar area after sacroplasty

**Fig. 2:**
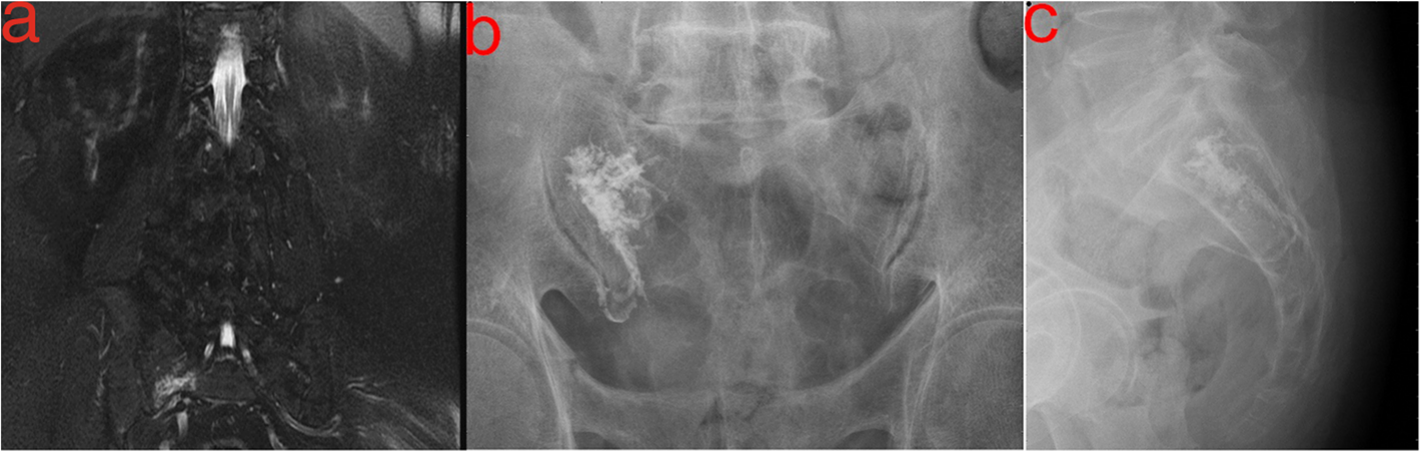
**a** Coronal section of spinal magnetic resonance imaging (MRI) indicating sacral insufficiency fracture involving the right alar area in an 81-year-old male patient. **b** Sacral lateral view of cement filling from S1 to S3 levels after sacroplasty. **c** Sacral anteroposterior view of cement filling in the right alar area after sacroplasty

Sacral insufficiency fractures (SIFs), first reported by Lourie in 1982 [1], can cause severe axial lumbosacral pain [2]. Patients with underlying conditions such as primary osteoporosis, prolonged steroid use, primary bone tumors, and metastatic disease have a higher risk of SIFs [1]. The prevalence of SIFs is 1–5% in at-risk populations [3, 4]. SIF is usually diagnosed on the basis of bone scintigraphy or magnetic resonance imaging (MRI) findings (Figs. 1a and 2a). Typically, these image findings present an H-pattern or the so-called Honda sign [5]. However, this H-pattern is reported in only 20–40% of patients with SIFs [6]. Unilateral or bilateral fractures in the sacral ala [7] with or without sacral body involvement, transverse fractures in the lower sacrum [8], and patterns with multiple foci [9] are variations in the image pattern of SIF.

Conservative treatments such as bedrest, rehabilitation, and analgesics are recommended for pain due to SIF [10]. Percutaneous sacroplasty is a minimally invasive treatment used to resolve persistent symptoms or severe pain caused by SIF (Figs. 1b, 2b, and c). Sacroplasty was first reported in 2002 [11], and the procedure relieved pain and improved mobility [12, 13]. Several case series with different techniques have been reported in the literature [14, 15].

Needle-insertion techniques for sacroplasty include the transiliac [16] (lateral approach under computed tomography [CT] or fluoroscopic guidance), long-axis [17] (usually under CT guidance), and short-axis [18] (under CT or fluoroscopy guidance) approaches. No consensus has been reached on the optimal sacroplasty approach; the method employed is at physicians’ discretion.

Needle insertion in alar sacroplasty is often performed under CT guidance due to the relatively complex anatomy of the sacrum combined with its multiple associated foramina containing sacral nerve roots [14, 19, 20]. Greater radiation exposure under CT guidance can pose risks to patient and physician safety. Furthermore, CT use makes the procedure time intensive and complex, and sedatives might be required to ensure patient cooperation. Here, we report our preliminary experience with a combination of long- and short-axis alar sacroplasty techniques under fluoroscopic guidance for osteoporotic SIFs.

## Materials and methods

### Data statement

Sacroplasty was performed in accordance with the relevant guidelines and regulations of the Taiwanese government. This study was approved by the Institutional Review Board of E-Da Hospital (EMRP-108-139), and the patient consents were not required in this study.

### Study design

We retrospectively reviewed 68 patients with symptomatic osteoporotic SIFs who underwent sacroplasty at our hospital between January 2013 and February 2020. SIFs were documented using either CT or MRI. Patients with metastatic cancer involving the sacrum, displaced SIFs, or SIFs involving S1 or S2 body or those who underwent balloon-assisted sacroplasty were excluded. The remaining 44 patients were divided into two groups: the study group (19 patients who received a combination of long- and short-axis alar sacroplasty techniques under fluoroscopic guidance) and the control group (25 patients who received short-axis alar sacroplasty under fluoroscopic guidance).

### Alar sacroplasty technique

**Fig. 3:**
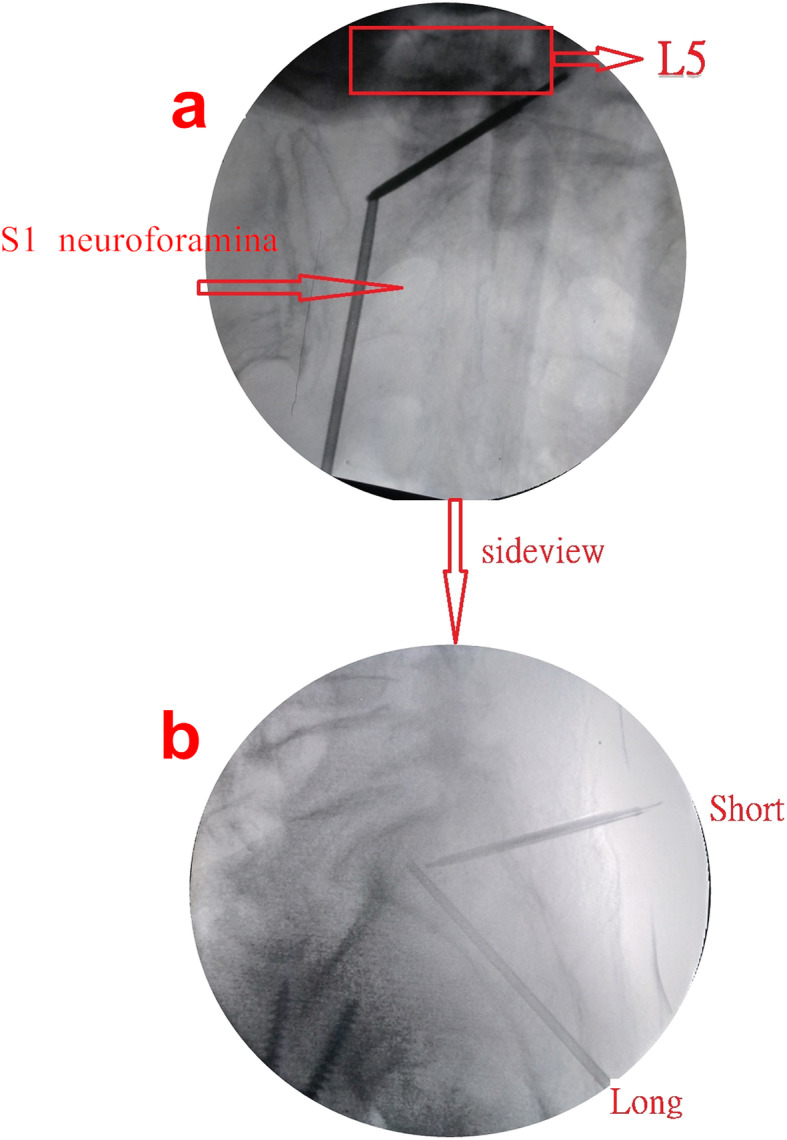
**a** Sacral anteroposterior view under fluoroscopy depicting a combination of long- and short-axis needles placed in the right alar area. **b** Sacral lateral view under fluoroscopy depicting a combination of long- and short-axis needles placed in the right alar area

The patients were placed in prone position during this procedure. Under conscious sedation with minor sedatives and local anesthesia, two bone biopsy needles (most commonly 11 gauge) were inserted under fluoroscopic guidance (Fig. 3 a and b). The C-arm was placed at an angle of 15–30° in cephalic direction to obtain an anteroposterior (AP) view of the sacrum.

**Fig. 4:**
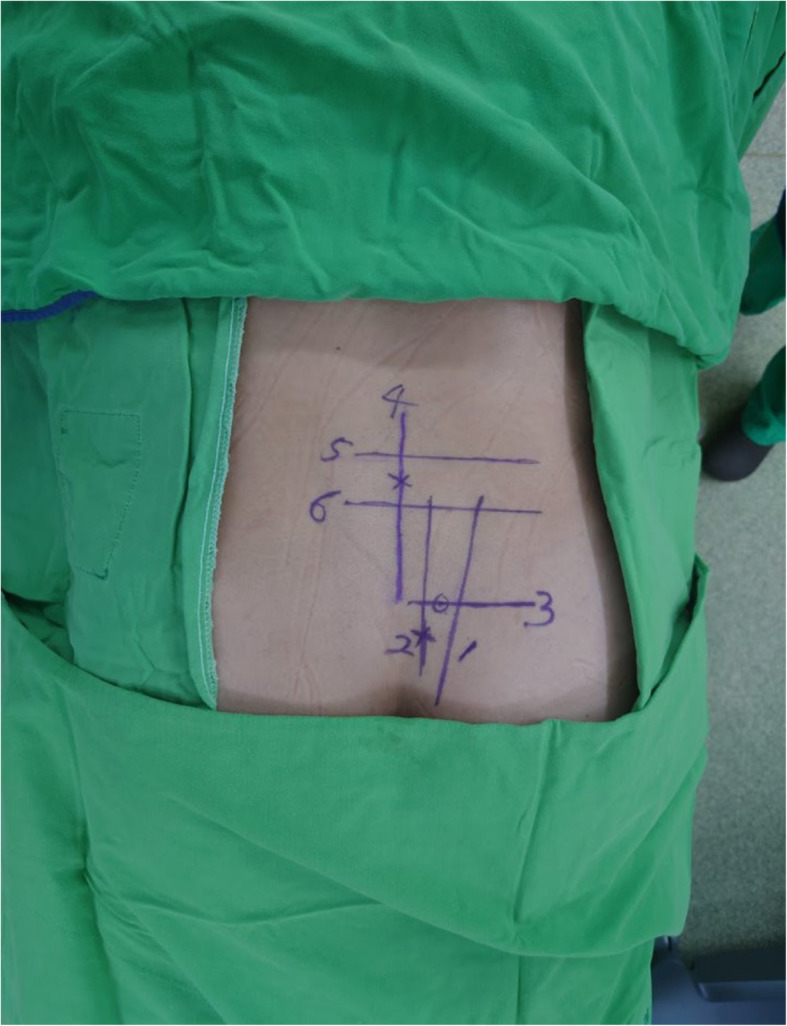
Skin landmarks on a patient’s back. 1, sacroiliac (SI) joint line; 2, line linking the lateral walls of S1 and S2 neuroforamina; 3, horizontal line at the tip of sacral ala; 4, vertical line parallel to the medial wall of the L5 pedicle; 5, line at the upper endplate of the S1 body; 6, horizontal line parallel to the upper wall of the S1 neuroforamen

**Fig. 5:**
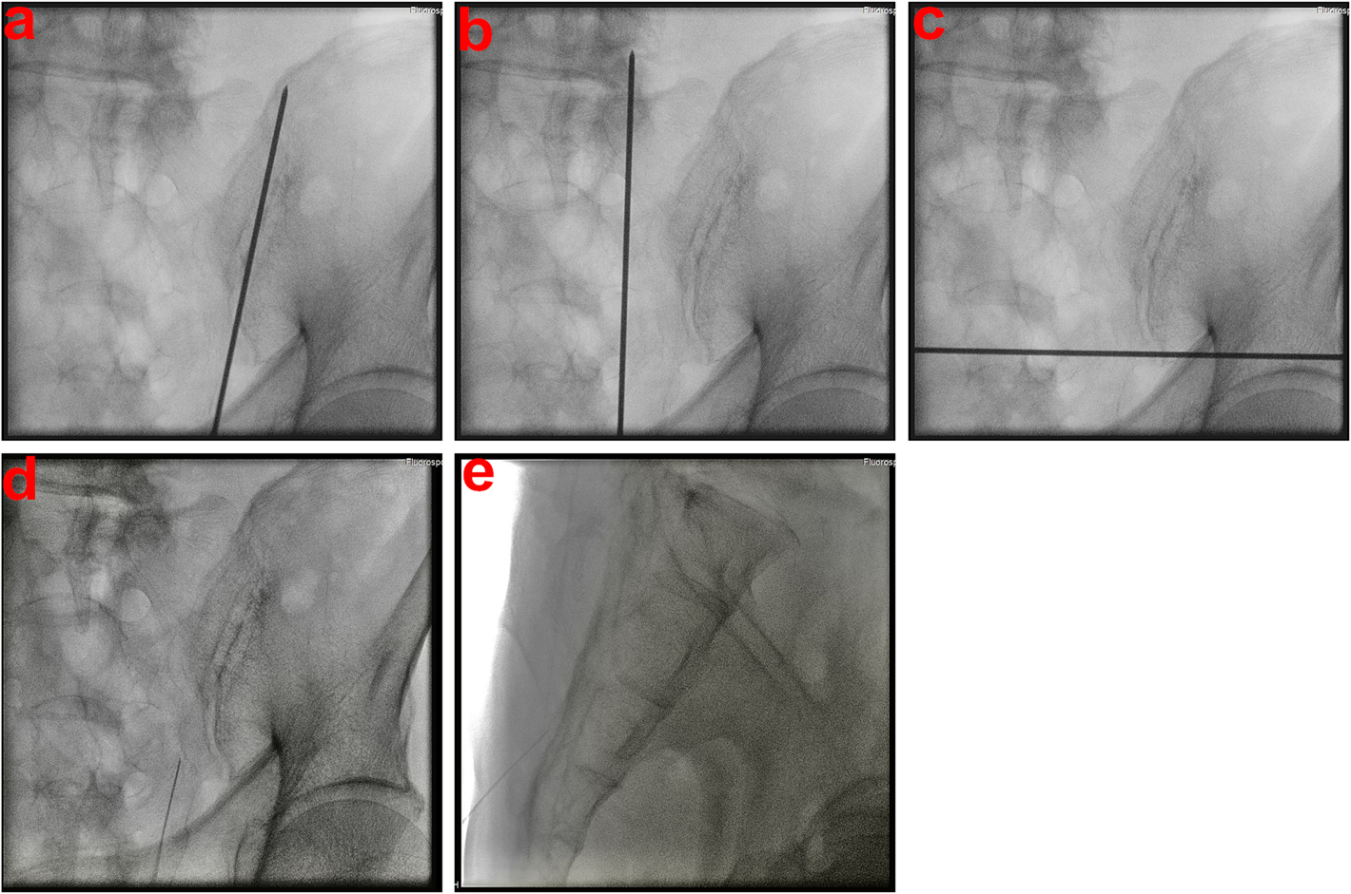
**a** Sacral anteroposterior view under fluoroscopy showing the sacroiliac (SI) joint line identified with a k-wire in the right alar area. **b** Sacral anteroposterior view under fluoroscopy showing a line linking the lateral walls of S1 and S2 neuroforamina identified with a k-wire in the right alar area. **c** Sacral anteroposterior view under fluoroscopy showing a horizontal line at the tip of the sacral ala identified with a k-wire in the right alar area. **d** Sacral anteroposterior view under fluoroscopy depicting a bony entry point of long-axis needles in the right alar area. **e** Sacral lateral view under fluoroscopy depicting a bony entry point of long-axis needles in the right alar area

Before needle insertion, bony landmarks in the AP view, including the L5 pedicle, S1 and S2 neuroforamina, sacral alar area, and sacroiliac (SI) joint, and in the lateral view, including the sacrum and L5-S1 disc, were identified on the skin of the patients’ backs (Fig. 4). These lines included the SI joint line (Fig. 5a), lines linking the lateral walls of S1 and S2 neural foramina (Fig. 5b), and a horizontal line at the tip of sacral ala (Fig. 5c) for long-axis needle insertion. The starting point of needle insertion on the skin could be located on the line linking the lateral walls of S1 and S2 neuroforamina at approximately 1 cm below the horizontal line drawn at the tip of the sacral ala (Fig. 4).

**Fig. 6:**
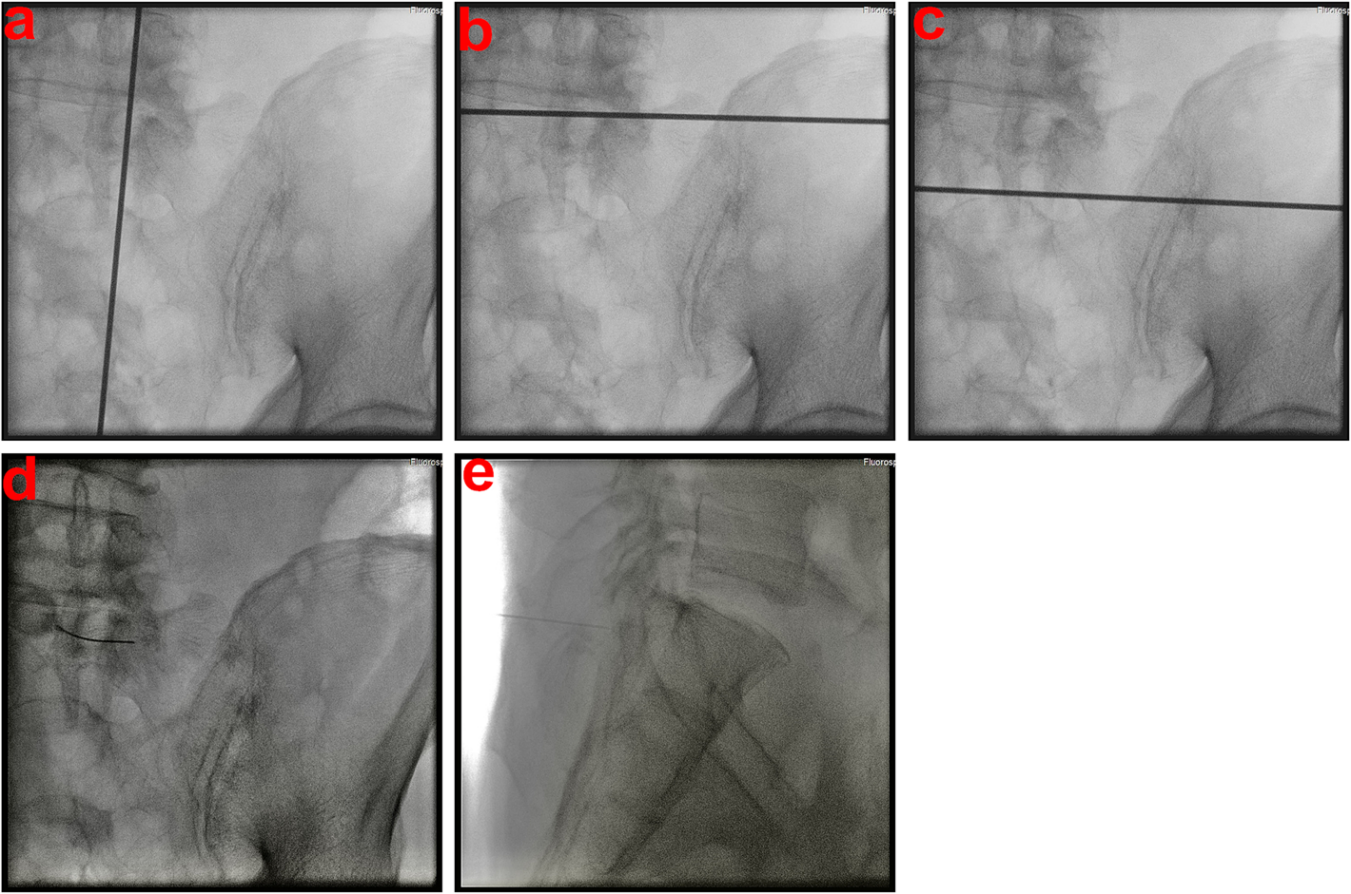
**a** Sacral anteroposterior view under fluoroscopy showing a vertical line parallel to the medial wall of the L5 pedicle identified with a k-wire in the right alar area. **b** Sacral anteroposterior view under fluoroscopy showing a line at the upper endplate of the S1 body identified with a k-wire in the right alar area. **c** Sacral anteroposterior view under fluoroscopy showing a horizontal line parallel to the upper wall of the S1 neuroforamen identified with a k-wire in the right alar area. **d** Sacral anteroposterior view under fluoroscopy depicting a bony entry point of short-axis needles in the right alar area. **e** Sacral lateral view under fluoroscopy depicting a bony entry point of short-axis needles placed in the right alar area

We made other skin landmarks on the patient’s back for short-axis needle insertion, and they included a vertical line parallel to the medial wall of the L5 pedicle (Fig. 6a), a line at the upper endplate of the S1 body (Fig. 6b), and a horizontal line parallel to the upper wall of the S1 neural foramina (Fig. 6c) in the AP view. The starting point on the skin could be located on the vertical line parallel to the medial wall of the L5 pedicle in the midpoint between the line at the upper endplate of S1 and the horizontal line parallel to the upper wall of the S1 neuroforamina (Fig. 4).

The entry point for long-axis needle insertion was at the center of the ala between the neuroforamen and SI joint at S3 in the AP and lateral views (Fig. 5 d and e), and the track of bone biopsy needle insertion from the skin starting point to the bony entry point could be checked by a small long needle during local anesthetic injection. The target point of needle insertion was the center of the ala at the S1 level in the AP view and in the anterior half of the S1 endplate in the lateral view (Fig. 3a). The long-axis needle was placed in an oblique orientation to bisect the ala (Fig. 3b).

After the long-axis needle insertion, the short-axis needle was inserted. The bony entry point was 0.5 cm above and lateral to the vertical line parallel to the medial wall of the L5 pedicle and the horizontal line parallel to the upper wall of the S1 neuroforamina in the AP view and dorsal cortex of S1 in the lateral view (Fig. 6 d and e), and the track of bone biopsy needle insertion from the skin starting point to the bony entry point could be checked by a small long needle during local anesthesia drug injection. The target point of needle insertion was identical to that of long-axis needle insertion, but the depth of short-axis needle insertion was shallower than the target point after the short-axis needle penetrated the dorsal cortex of the sacrum (Fig. 3 a and b).

Prior to cement injection, a central metallic bar with an obtuse tip was inserted through the cannula to confirm the absence of obstructions. The bar was also used to sound the surrounding environment to ensure that the needle was located inside the cancellous bone within the cortical wall boundary. Then, the cement was injected into the sacral ala under real-time fluoroscopy (Fig. 2) by using a 10-mL syringe with cement injection equipment, which maintained a slow continuous rate of injection. The goal of the injection was to fill the fracture site and as much of the surrounding bone as possible. Warning bony marks for avoiding cement extravasation were the lateral wall of the neural foramina and SI joint in the AP view and the dorsal cortex of the sacrum and S1 endplate in the lateral view. Cement injection was stopped when the cement reached the warning marks. After the cement was filled at the S1 level, the long-axis needle was retraced to the S2 and S3 levels, and as much cement was injected as possible.

After the procedure, patients were observed in a postoperative observation room. Patients were usually discharged on the next day after sacroplasties. After discharge, patients were followed up at the outpatient department at 1, 2, 6, and 12 months. Preoperative and postoperative visual analog scale (VAS) for pain scores was recorded. Clinical symptoms and adverse events were also recorded.

## Results

The study group comprised 3 male and 16 female patients (mean age, 77.37 years). The control group comprised 4 male and 21 female patients (mean age, 81.2 years). The patients’ baseline characteristics are listed in Table 1. The mean follow-up duration was 12.84 and 36.12 months in the study and control groups, respectively. All patients reported symptom improvement after alar sacroplasty. The mean VAS pain score decreased from 8.42 preoperatively to 1.47 postoperatively in the study group and from 8.72 preoperatively to 1.52 postoperatively in the control group. The mean bone mineral density (BMD) was −3.247 and −3.38 in the study and control groups, respectively. All patients received antiosteoporotic drugs after receiving a definite osteoporosis diagnosis based on BMD values.

**Table 1:** Baseline characteristics of patients

	Combine (*N*=19)	Short axis (*N*=25)	*P* value
Age	77.37±8.45	81.2±5.83	0.123
BMD	−3.247±0.54	−3.38±0.73	0.524
Preoperative pain score	8.42±0.51	8.72±0.46	0.048
Postoperative pain score	1.47±0.61	1.52±0.57	0.746
Gender			0.999
Female	16 (84.2)	21 (84)	
Male	3 (15.8)	4 (16)	
Cement volume	3.55±0.96	2.94±0.89	0.074
op time (min)	32±7.1	28.04±4.99	0.046
Follow-up (months)	12.84±10.02	36.12±19.17	*P<0.001*
Other level of osteoporotic compression fracture	15 (78.9)	23 (92)	0.378

The mean volume of injected cement was 3.55 ± 0.96 mL (range 2.5–5 mL) in the study group and 2.94 ± 0.89 mL (range 1.5–4 mL) in the control group. The injected cement volume did not differ significantly between the two groups. The mean operation time was 32 ± 7.1 min (range 17–40 min) in the study group and 28.04 ± 4.99 min (range 20–40 min) in the control group (*P* = 0.046).

Four cases of cement leakage occurred: three at the S1 neuroforamina (one in the study group and two in the control group) and one at the caudal alar tip at the S3 level. Fortunately, no clinical symptoms of cement leakage were present.

No incidence of surgical infection after the sacroplasty procedures or recurrent pain due to SIFs was reported during the follow-up period.

## Discussion

Percutaneous vertebroplasty has become an increasingly common treatment option for osteoporotic vertebral compression fractures since it was introduced by Galibert et al. in 1987 [21]. This technique is used for cylindrical vertebral bodies and shield-shaped sacral bones [22]. Cement injection in osteoporotic vertebral fractures can offer mechanical stability, which prevents painful micromotion, restores pelvic strength, and has analgesic effects due to its thermal properties [11, 15, 23].

Several studies have reported that percutaneous sacroplasty can be an effective alternative strategy to conservative therapy for SIFs [12, 22, 24–27]. Pain relief with decreased VAS scores for pain and functional recovery can be expected after percutaneous sacroplasty [16]. Our study showed similar results, with reduced VAS scores for pain in both groups.

The technique for alar sacroplasty is similar to that for vertebroplasty with some differences. One challenge of this technique is determining when the needle tip has reached the inner cortical margin of the sacrum but has not traversed into the pelvic side. Furthermore, identifying sacral foramina under only fluoroscopic guidance might be difficult [16]. Needle placement in the sacral foramina prior to cement injection was reported to prevent cement leakage to the sacral foramina [16]. For our patients, we placed the C-arm at an angle of 15–30° to obtain the AP view of the sacrum to improve the visualization of the sacral foramina.

Some case reports have indicated that alar sacroplasty under CT guidance achieves more accurate positioning of bone biopsy needles [28]. CT use, however, enables the delivery of a higher radiation dose to the patient and increases radiation exposure to surgeons during needle insertion into the sacrum [16]. As a solution to these technical limitations, fluoroscopy during sacroplasty has been suggested in some reports as it has lower radiation exposure for both patients and surgeons [16].

The most crucial concern in alar sacroplasty under fluoroscopic guidance is ensuring that the needle is inserted precisely into the target area. According to Jayaraman et al., a safe target for sacroplasty needle placement in the superolateral sacral alar is the point of intersection of lines drawn from each corner of the S1, which is readily identifiable with lateral fluoroscopy [29]. Whitlow et al. reported that for the safe placement of a short-axis injection needle under CT guidance, the following must be ensured: the needle is parallel to the L5–S1 interspace and ipsilateral SI joint and targeting the superolateral sacral ala within an area bounded by a line lateral to the posterior foraminal openings and a line superimposed on the medial edge of the SI joint [30]. CT guidance is usually needed while performing long-axis needle insertion [17].

In the present study, we chose the same target in the superolateral sacral ala at the S1 level. The long-axis needle reached the target, and the short-axis needle was inserted at a shallow depth. All procedures were performed under fluoroscopic guidance. We determined that alar sacroplasty with combined short- and long-axis needles can be safely performed under fluoroscopy. The combined short- and long-axis needles can provide the pressure required to release and drain fluid accumulated in SIFs to facilitate smooth cement injection. The advantage is similar to that of the bipedicle approach of vertebroplasty for osteoporotic vertebral compression fractures [31].

We compared the operation time and injected cement volume between the two groups. The operation time was significantly longer in the study group (32 ± 7.1 vs 28.04 ± 4.99 min; *P* = 0.046). The injected cement volume may have been higher in the study group, but the difference was nonsignificant.

Our study has some limitations. It included a small number of patients, and although complications of the technique did not transpire, they have a chance of occurring. For instance, cement leakage problems could occur, leading to major diastasis. Some previous studies have reported that balloon-assisted sacroplasty [32, 33] and using high-viscosity cement [34, 35] are suitable options for preventing cement leakage. Future studies on monitoring and preventing adverse events are required.

In conclusion, a combination of long- and short-axis alar sacroplasty techniques can be effectively performed under fluoroscopic guidance for osteoporotic SIFs.

## Abbreviations

SIF: Sacral insufficiency fracture
VAS: Visual analog scale
MRI: Magnetic resonance imaging
CT: Computed tomography
AP: Anteroposterior
SI: Sacroiliac
BMD: Bone mineral density
